# Permanent Pacemaker Implantation in a Patient with Takotsubo Cardiomyopathy and Complete Atrioventricular Block

**DOI:** 10.1155/2021/6637720

**Published:** 2021-04-02

**Authors:** Toshihiro Terui, Masumi Iwai-Takano, Tomoyuki Watanabe

**Affiliations:** ^1^Division of Internal Medicine, Health Co-op, Watari Hospital, Fukushima, Japan; ^2^International Community Health, Graduate School of Medicine, Fukushima Medical University, Fukushima, Japan; ^3^Department of Neuropsychiatry, Fukushima Medical University, Fukushima, Japan; ^4^Department of Epidemiology, Fukushima Medical University, Fukushima, Japan; ^5^Division of Cardiovascular Surgery, Fukushima Medical University, Fukushima, Japan; ^6^Fukushima Prefectural General Hygiene Institute, Fukushima, Japan

## Abstract

This case report presents a patient with Takotsubo cardiomyopathy (TCM) and complete atrioventricular (AV) block who was treated with permanent pacemaker implantation. A 78-year-old woman with a history of hypertension presented with a 6-month history of palpitations. On initial evaluation, her heart rate was 40 beats/minute. Electrocardiography revealed a complete AV block and T-wave inversion in these leads: I, II, aVL, aVF, and V3–6. Echocardiography showed akinesis from the midventricle to the apex and hyperkinesis on the basal segments. The patient was diagnosed with TCM and complete AV block. Because improvement of TCM may subsequently improve the AV node dysfunction associated with TCM, the patient was admitted for treatment of heart failure without pacemaker implantation. The left ventricular (LV) abnormal wall motion improved gradually; however, the AV block persisted intermittently. On hospital day 14, a pause of 5–6 seconds without LV contraction was observed, and permanent pacemaker implantation was performed. On day 92, echocardiography revealed normal LV wall motion. However, electrocardiography revealed that the pacemaker rhythm with atrial sensing and ventricular pacing remained. Although specific degree of damage that may result from AV block associated with TCM is unknown, some of these patients require pacemaker implantation, despite improvement of abnormality in LV wall motion.

## 1. Introduction

Takotsubo cardiomyopathy (TCM) is known as stress-induced heart disease that occurs with transient acute left ventricle (LV) apical ballooning without coronary artery stenosis. Various pathogeneses of TCM are presumed, and strong emotional or physical stress has been reported to be triggers [1]. Various types of arrhythmias can complicate acute phase TCM, such as ventricular tachycardia, ventricular fibrillation, Torsades de pointes, atrial fibrillation, sinus node dysfunction, and atrioventricular (AV) block [1–3]. Contrary to the reversible, transient pathophysiology of TCM alone, some cases of arrhythmia-complicated TCM developed into more life-threatening conditions, such as intermittent asystole or sudden cardiac death [2]. However, only a few reports have described TCM patients with AV block who underwent pacemaker implantation. Among those reports, AV node dysfunction remained even after LV contraction was improved [4–6]. However, AV node conduction normalized for some patients over a long-term follow-up period [7]. Because the specific degree of damage that occurs in AV conduction in the setting of TCM is unknown, the best method for the management of advanced AV block associated with TCM remains unclear [8]. This case report presents a patient with persistent AV block-complicated TCM, the treatment of which was permanent pacemaker implantation in the process of improving LV dysfunction.

## 2. Case Presentation

A 78-year-old woman with hypertension complained a 6-month history of palpitations and presented to her local clinic, where physical examination showed bradycardia. She was then referred to the authors' hospital for further evaluation.

**Figure 1:**
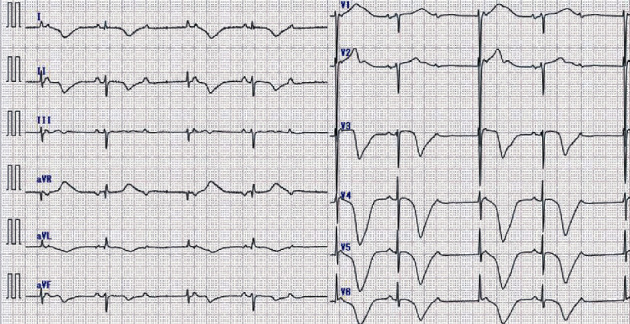
The initial electrocardiogram in the emergency department shows complete atrioventricular block with a heart rate of 40 beats/minute and T-wave inversion in the I, II, aVL, aVF, and V3–6 leads.

On physical examination, blood pressure was 153/78 mmHg, heart rate was 46 beats/minute, and peripheral capillary oxygen saturation was 96% on room air. Chest radiography showed enlargement of the cardiothoracic ratio and blunting of the right costophrenic angle. Initial electrocardiography revealed complete AV block with a junctional escape rate of 40 beats/minute, and T-wave inversion in these leads: I, II, aVL, aVF, and V3–6 (Figure 1). Laboratory data showed elevations of troponin T 184 ng/L (normal, <50 ng/L) and brain natriuretic peptide 287.5 pg/mL (normal, <18.4 pg/mL), although serum creatine kinase was normal. Echocardiography showed akinesis from the midventricle to the apex of the LV and hyperkinesis on the basal segments. Mild mitral regurgitation and mild tricuspid regurgitation without elevated velocity of tricuspid regurgitation were also observed.

**Figure 2:**
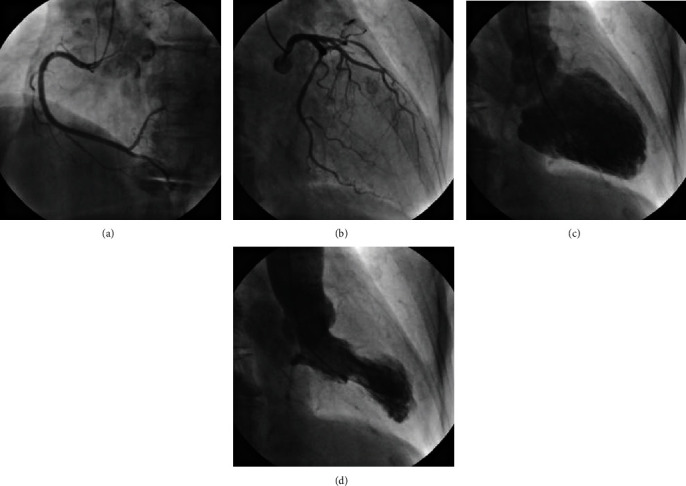
On hospital day 8, a coronary angiogram shows no stenosis: (a) right coronary artery; (b) left coronary artery. Left ventricular angiography shows hypokinesis from the midventricle to the apex and hyperkinesis in the basal segments: (c) end-diastole; (d) end-systole.

The patient was diagnosed with TCM and complete AV block. Because AV node dysfunction associated with TCM may be recovered after improvement of TCM, she was conservatively treated for heart failure without pacemaker implantation. On hospital day 8, coronary angiography showed no significant stenosis, and left ventriculography revealed apical ballooning (Figure 2).

**Figure 3:**
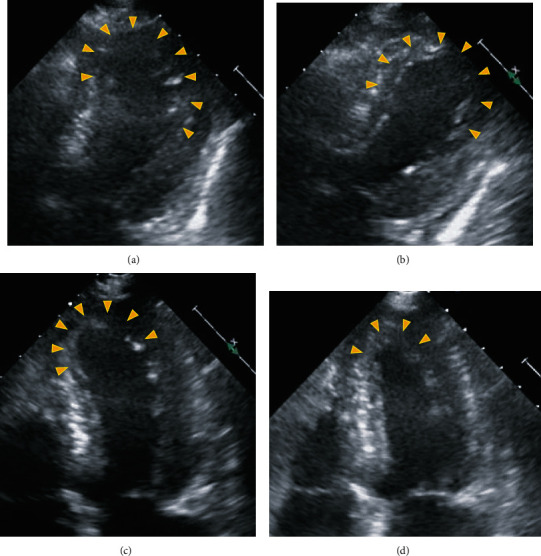
Echocardiogram shows gradual improvement of left ventricular systolic dysfunction: (a) admission (day 0), (b) hospital day 2, (c) day 5, and (d) day 9. Yellow arrow indicates an area of abnormal wall motion in end-systole.

**Figure 4:**
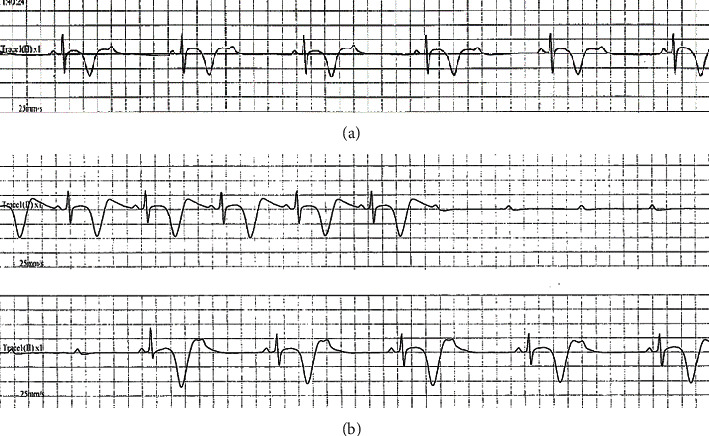
Electrocardiogram shows transient advanced atrioventricular block on hospital day 2 (a) and day 14 (b).

Echocardiography showed improvement of LV abnormal wall motion gradually over admission through hospital day 9 (Figure 3); however, AV block persisted intermittently (Figure 4(a)). On day 14, a pause of 5–6 seconds without LV contraction was observed (Figure 4(b)), and the patient underwent temporary pacemaker implantation. On day 15, a permanent pacemaker was implanted.

On day 92, echocardiography revealed normal LV wall motion. However, electrocardiography showed that the pacemaker rhythm (DDD mode, AV delay 180 msec) with atrial sensing and ventricular pacing remained.

## 3. Discussion

The occurrence of AV block is rarely associated with TCM, with a prevalence of about 2.9% [2]. Previous AV block and its intervention, such as temporary or permanent pacemaker implantation, can be triggers of myocardial dyskinesis [9, 10]. Subsequently, myocardial abnormalities such as fibrosis or edema have the pathologic potential to create ventricular electrical stimuli. However, the definitive demonstration of a cause/effect relationship between myocardial edema and electrical abnormalities, not only in TCM but also in other conditions of reversible ventricular dysfunction, is still lacking [11]. Thus, it remains unknown whether TCM causes AV block or AV node dysfunction as complications or conversely whether AV block as a physical stressor causes TCM.

Patient's age may be a contributor to AV dysfunction, and AV block may be more common in older adult women. [12]. In the current case, a 78-year-old woman experienced palpitations before developing TCM. It was expected that the AV block would recover along with improvement of LV systolic dysfunction; however, the AV block remained despite improvement of the abnormality in LV wall motion. Thus, the hypothesis was that previous AV node dysfunction, which progressed with aging, induced the TCM in this patient. Moreover, 3 months after permanent pacemaker implantation, echocardiography showed normal LV wall motion, but the patient's AV block persisted.

It remains unclear whether permanent pacemaker implantation in patients with AV block complicated by TCM is an appropriate treatment, and it is not possible to predict proper implantation in the acute phase of TCM. Some reports describe patients who underwent permanent pacemaker implantation for AV block-complicated TCM on day 1 to day 18 [8–10, 12–14]. However, AV block has been reported to improve in the acute phase of TCM (on day 3) in some cases [7], and the recovery of AV node dysfunction 3 months after permanent pacemaker implantation in a patient with TCM has also been reported [15]. In contrast, AV block remained in several reported patients after improved LV wall motion abnormality in the chronic phase of TCM [8, 14]. Several investigators have suggested that permanent pacemaker implantation in the acute phase of TCM is required in these patients with AV block because of the persistence of AV block in this setting and the prognosis [16, 17].

Because the specific degree of damage of AV conduction in TCM is unknown, the treatment strategy for AV block in TCM patients remains unclear. However, some cases require permanent pacemaker implantation for advanced AV block despite improvement of LV wall motion abnormality.

## 4. Conclusion

Permanent pacemaker implantation was performed in a patient with AV block-complicated TCM. Some cases require permanent pacemaker implantation for advanced AV block, despite improvement of LV wall motion abnormality.

## Data Availability

The data used to support this study are included within the text of this report as well as in the cited references.
